# Supplementary material for: The causal relationship between the human gut microbiota and pyogenic arthritis: a Mendelian randomization study
Source: Front Cell Infect Microbiol. 2024 Nov 26;14:1452480. doi: 10.3389/fcimb.2024.1452480 (PMC11629706; doi:10.3389/fcimb.2024.1452480)
Supplement: Supplementary file 1 [file DataSheet1.zip › mendelian test/ebi-a-GCST90027487.csv_leave_one_out.pdf]

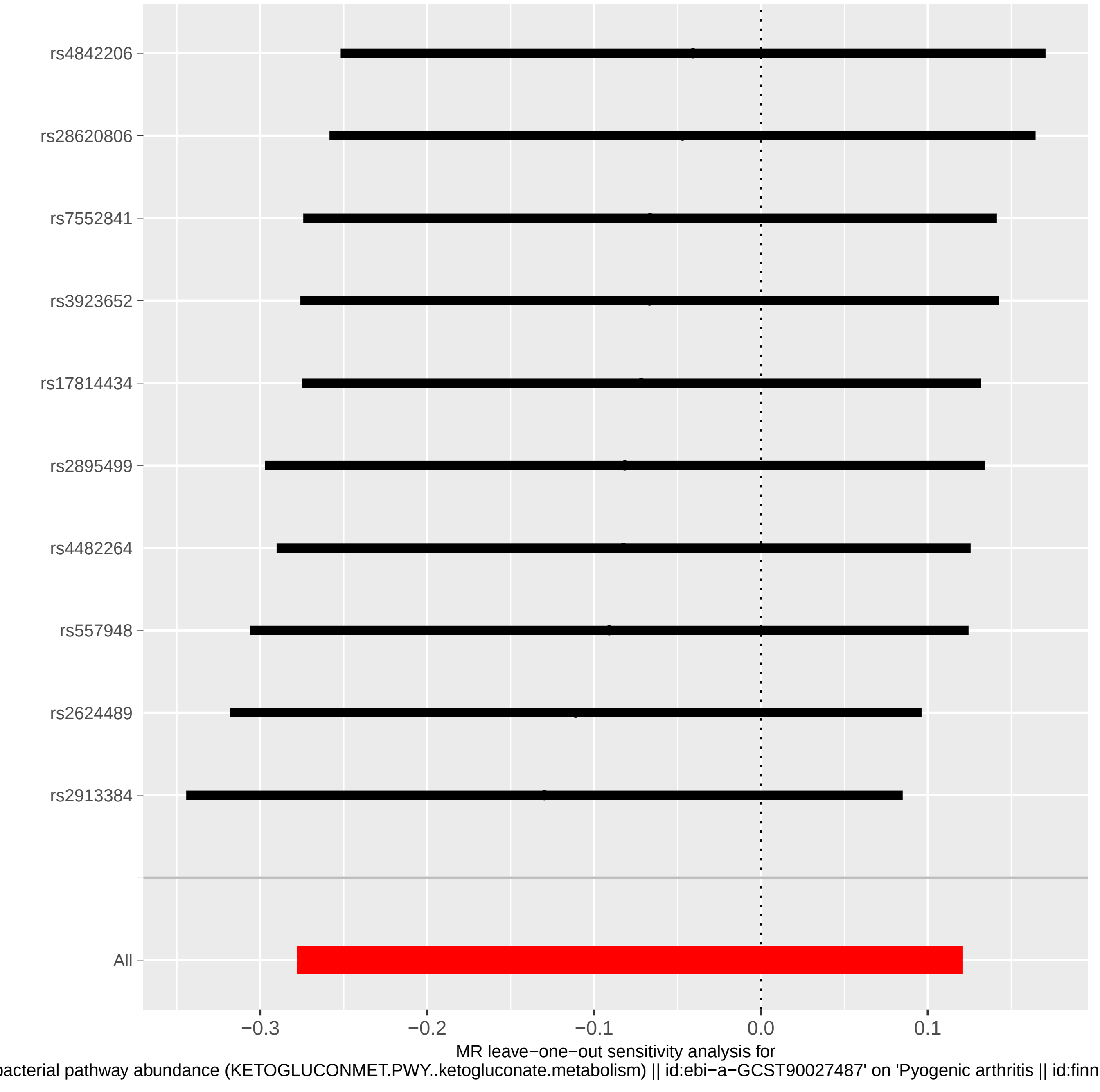

rs4842206
rs28620806
rs7552841
rs3923652
rs17814434
rs2895499
rs4482264
rs557948
rs2624489
rs2913384
All
−0.3
−0.2
−0.1
0.0
0.1
MR leave−one−out sensitivity analysis for
bacterial pathway abundance (KETOGLUCONMET.PWY..ketogluconate.metabolism) || id:ebi−a−GCST90027487' on 'Pyogenic arthritis || id:finn
